# Supplementary material for: CUT&Tag2for1: a modified method for simultaneous profiling of the accessible and silenced regulome in single cells
Source: Genome Biol. 2022 Mar 17;23:81. doi: 10.1186/s13059-022-02642-w (PMC8928696; doi:10.1186/s13059-022-02642-w)
Supplement: Supplementary file 1 — Additional file 1: Figure S1. Feature definition and fragment length separation under CUTAC conditions. Example tracks and length distributions of CUTAC data. Figure S2. Close correspondences between CUTAC and ATAC-seq. Heat maps, example tracks and correlation matrices comparing CUTAC and ATAC-seq. Figure S3. Determination of critical features for deconvolution by 2for1separator. Heat maps of 2for1separator output using different strategies. Figure S4. Single-Cell CUT&Tag2for1 and 2for1separator are consistent across replicates. UMAPs of Replicate data. Figure S5. Overlapping peaks. Heat maps of overlapping peaks. [file 13059_2022_2642_MOESM1_ESM.pdf]

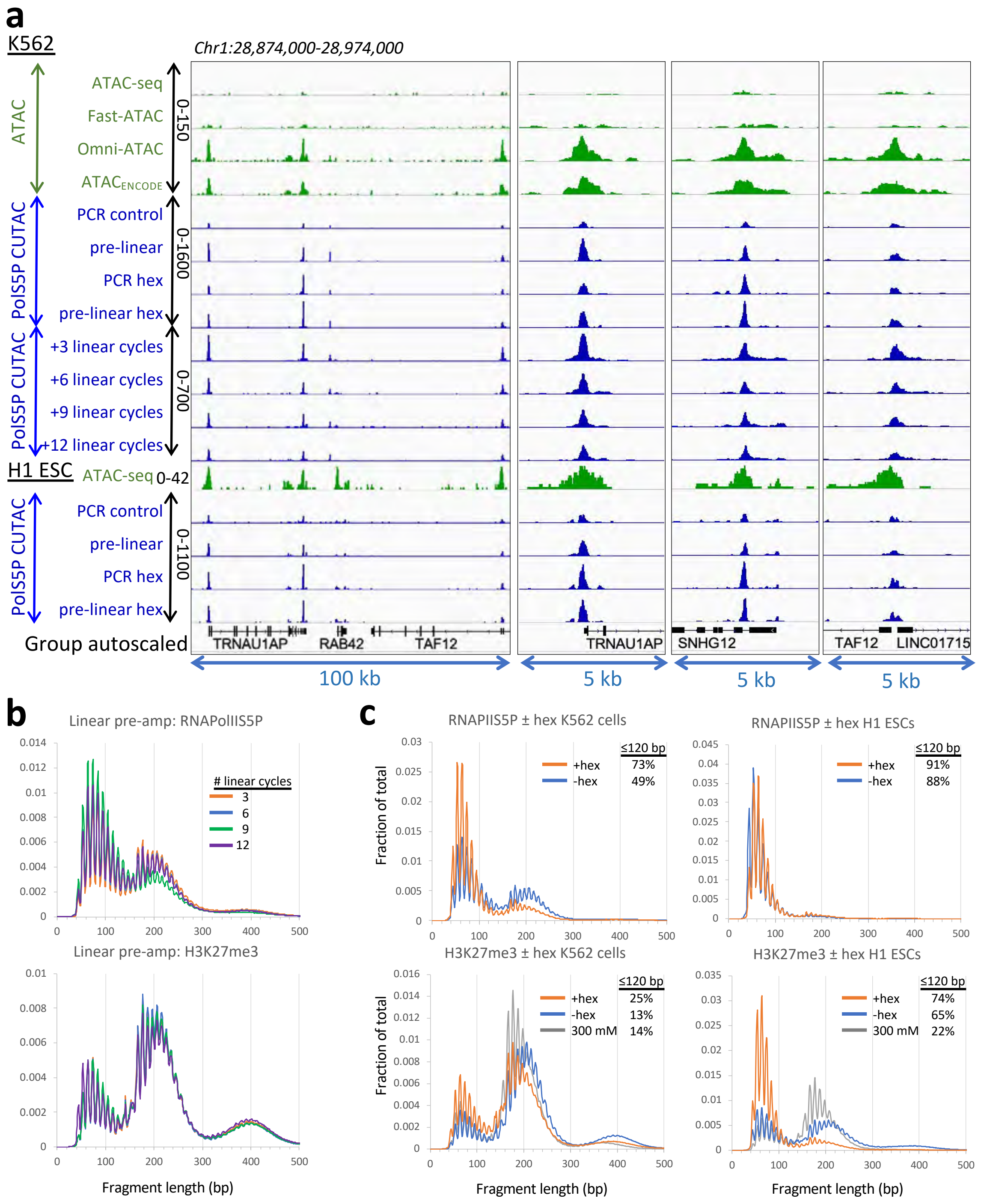

Additional file 1: Fig S1. Feature definition and fragment length separation under CUTAC conditions. (**a**) Fragments were mapped to hg19, and 3.2 million fragments were randomly sampled from each dataset and used to make bedgraph tracks. A representative region is shown. To compare peaks with very different signal-to-noise levels, samples were group-autoscaled with ranges indicated to the left of each set of tracks. Pol2S5p CUTAC of K562 cells with linear pre-amplification using only P5 primers for 12 cycles was followed by addition of P7 primers and PCR for various numbers of cycles. (**b**) Size distributions were not affected by differences in the number of PCR cycles following linear amplification. (**c**) Fragment length distributions for K562 cells (left) and H1 ES cells (right) are shown for linear pre-amplified fragments after tagmentations in 10 mM TAPS + 5 mM $MgCl_2$ ± 1,6-hexanediol or 300 mM NaCl + 10 mM $MgCl_2$ plotted as fractions of the total mapped fragments for linear pre-amplified datasets. Percentages of total fragments ≤120 bp iare shown. Tagmentation in 1,6-hexanediol generally results in a smaller fragment distribution, especially conspicuous for H1 cell nucleosomes. The higher recovery of nucleosome-sized fragments from K562 cells than H1 ES cells reflects the much lower abundance of Polycomb domains in H1 cells.

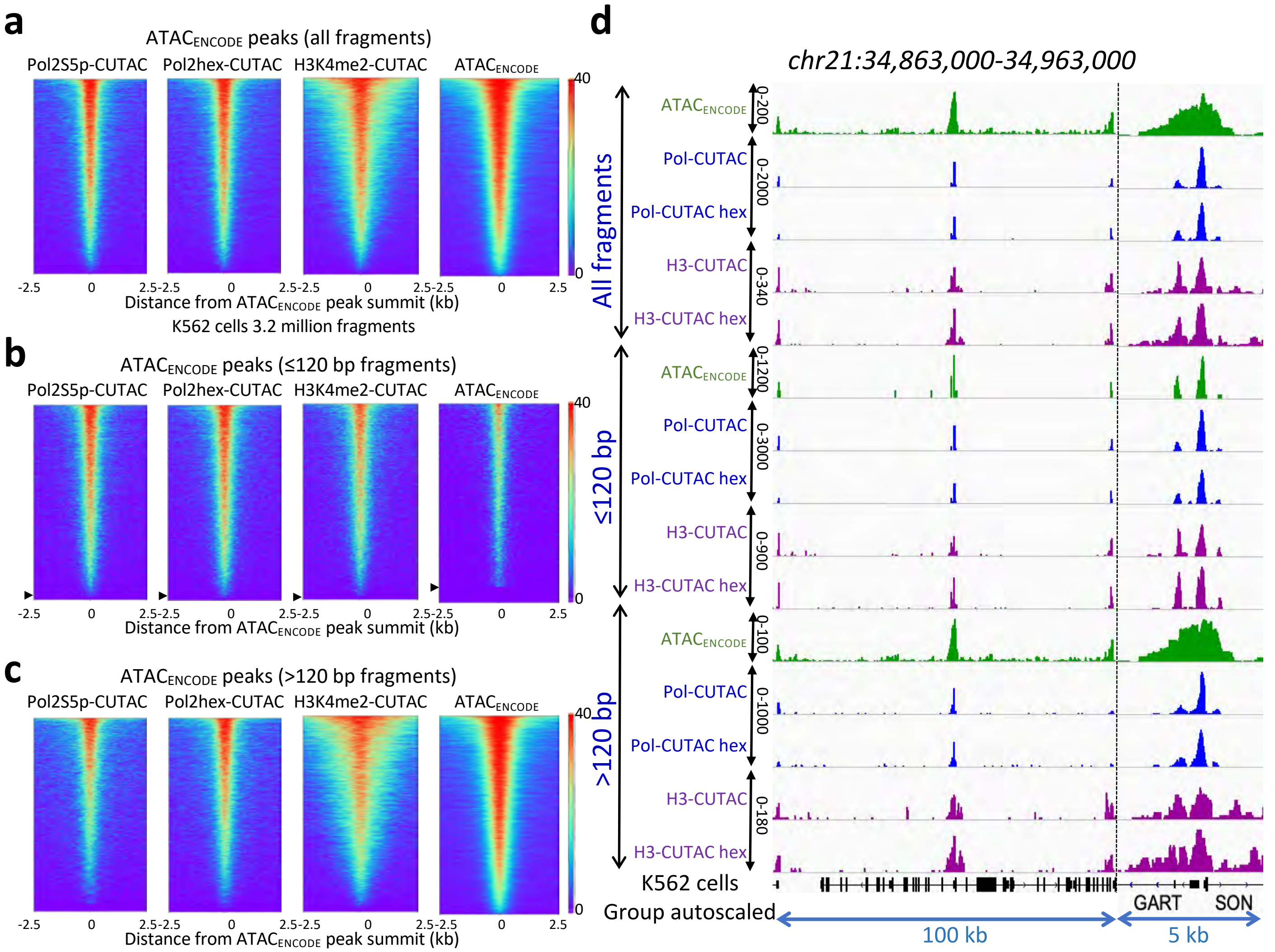

e

N=38155	ATAC_1	ATAC_2	H3-CUTAC	H3-CUTAC hex	Pol-CUTAC	Pol-CUTAC hex
**ATAC_1**	1	0.89	0.62	0.62	0.52	0.54
**ATAC_2**	0.89	1	0.61	0.62	0.52	0.53
**H3-CUTAC**	0.62	0.61	1	0.9	0.66	0.65
**H3-CUTAC hex**	0.62	0.62	0.9	1	0.58	0.58
**Pol-CUTAC**	0.52	0.52	0.66	0.58	1	0.96
**Pol-CUTAC hex**	0.54	0.53	0.65	0.58	0.96	1

R: 0, 0.5, 1

Additional file 1: Fig. S2. Close correspondences between CUTAC and ATAC-seq. (**a**) Heatmaps showing alignments of Pol2S5p-CUTAC ±10% 1,6-hexanediol during tagmentation, H3K4me2-CUTAC and $ATAC_{ENCODE}$ data over peaks called on an $ATAC_{ENCODE}$ replicated dataset. (**b**) Same as (a) showing the ≤120-bp fragment subset, where arrowheads indicate the row where enrichment of signal over peaks ends: 98-99% for CUTAC and 93% for $ATAC_{ENCODE}$. (**c**) Same as (b) showing the >120 bp subset. Fragments were mapped to hg19, and 3.2 million fragments were randomly sampled from each dataset and used to make bedgraph tracks. **(d**) Tracks comparing Pol2S5p and H3K4me2 CUTAC to ATAC-seq using K562 cell data generated by the ENCODE project. The region around the GART-SON bidirectional promoter is shown at two different scales. (**e**) Correlation matrix of CUTAC and ATAC-seq datasets used in this analysis. ATAC_1 is the ENCODE dataset used to call peaks and ATAC_2 is a replicate of ATAC_1.

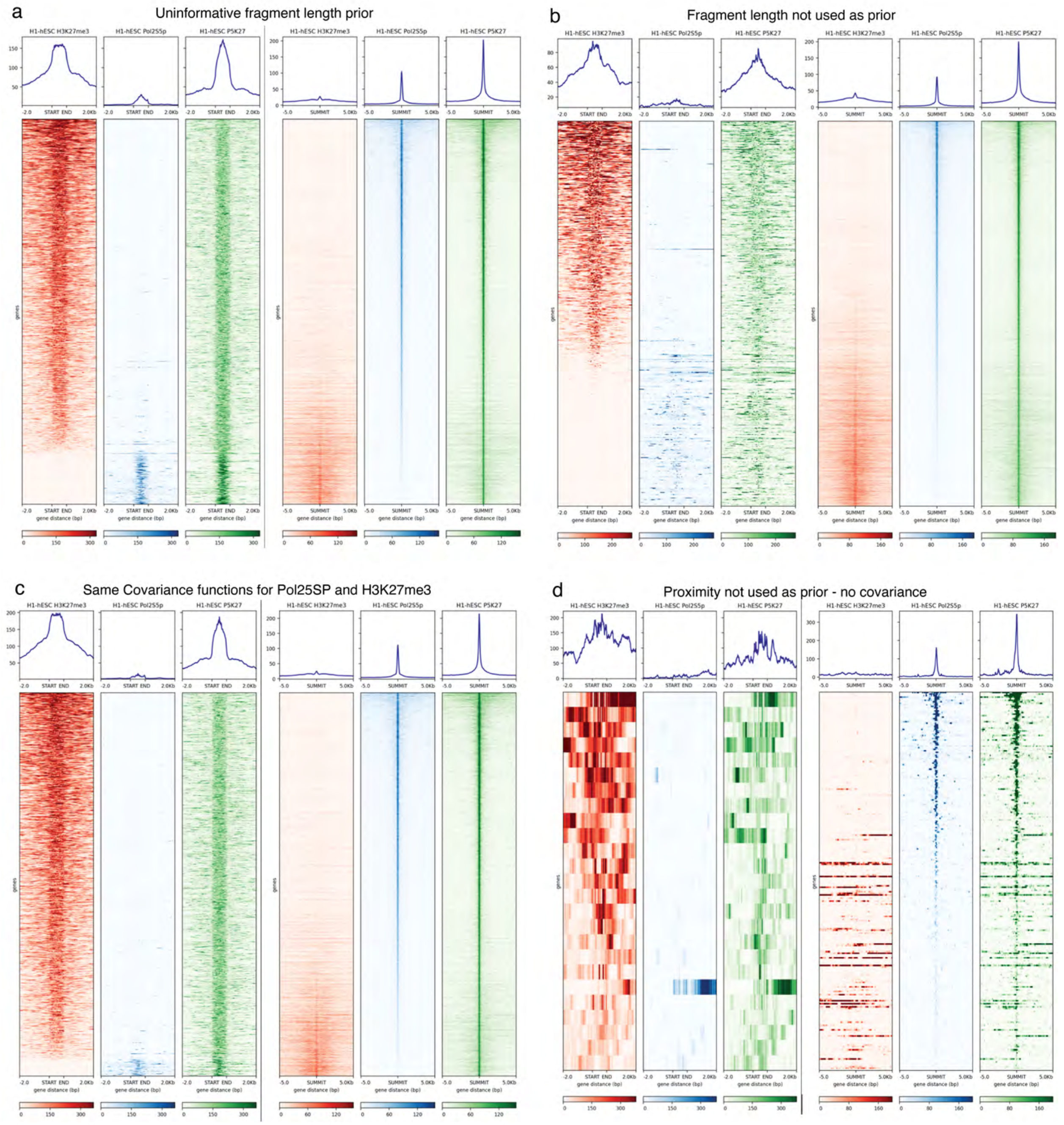

Additional File 1: Fig. S3. Determination of critical features for deconvolution by 2for1separator. (**a**) Deconvolution of H1 CUT&Tag2for1 data using an uninformative dirichlet prior (1, 1, 1, 1) for fragment length distribution. Left heatmaps show signal for H3K27me3 and right heatmaps show signals for Pol2S5P. Deconvolution is not dependent on prior knowledge about fragment size distributions. (**b**) Deconvolution without fragment length as prior leads to drop in performance. (**c**) Use of same covariance function for H3K27me3 and Pol2S5P does not degrade performance whereas not using proximity information leads to failure of denconvolution (**d**).

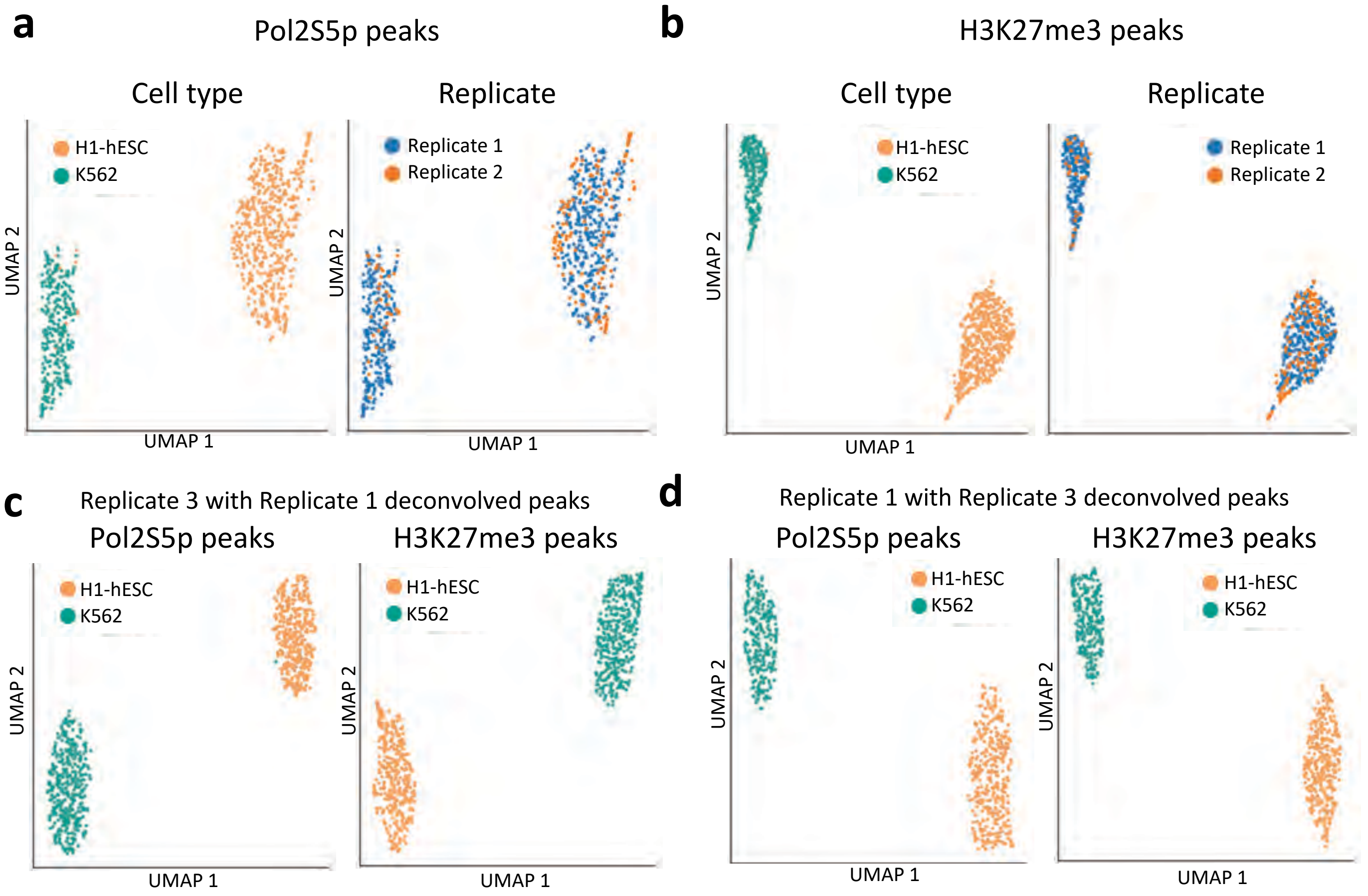

Additional file 1: Fig. S4. Single Cell CUT&Tag2for1 and 2for1separator are consistent across replicates. (**a**) UMAP plot showing that single K562 cells run as independent replicates cluster together according to their deconvolved Pol2S5p signal and away from the two replicates of H1-hESCs. (**b**) Same as (a) showing cells from two independent replicates clustered according to their deconvolved H3K27me3 signal. (**c**) UMAP showing that single cells from replicate 3 cluster according to the K562 and H1-hESCs cell types based on either the Pol2S5p peaks (left) or H3K27me3 peaks (right) deconvolved from replicate 1. (**d**) Same as (c) but showing the cells from replicate 1 plotted on the deconvolved peaks from Replicate 3.

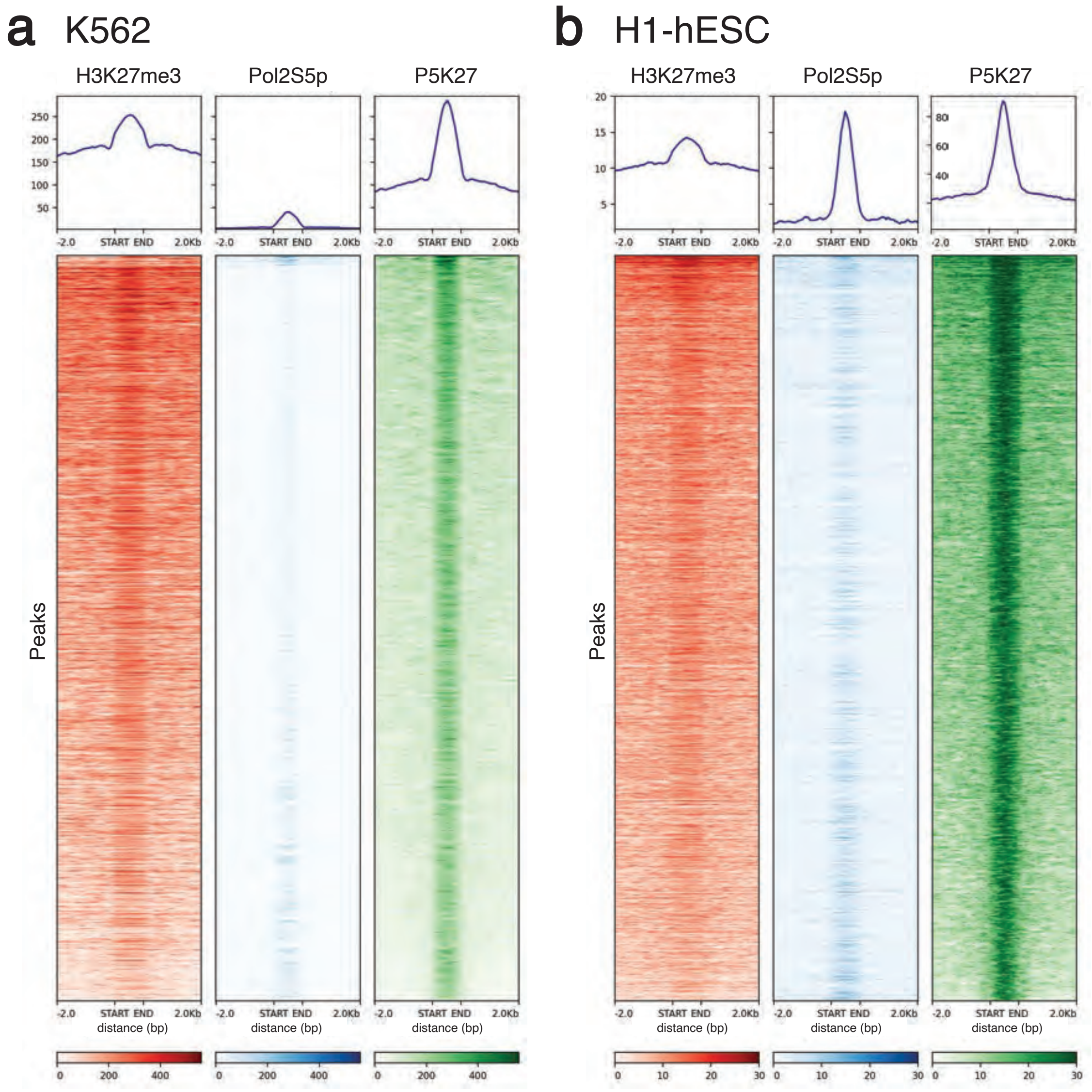

Additional file 1: Fig. S5. Overlapping peaks. (**a**) Single antibody and 2for1 data at the overlapping peaks for K562 cells. (**b**) Same as (a), for H1-hESC cells.
