# Supplementary material for: CUT&Tag2for1: a modified method for simultaneous profiling of the accessible and silenced regulome in single cells
Source: Genome Biol. 2022 Mar 17;23:81. doi: 10.1186/s13059-022-02642-w (PMC8928696; doi:10.1186/s13059-022-02642-w)
Supplement: Supplementary file 2 — Additional file 2. Review history. [file 13059_2022_2642_MOESM2_ESM.docx]

Review History

**First round of review**

**Reviewer 1**

**Were you able to assess all statistics in the manuscript, including the appropriateness of statistical tests used?**

Yes: No additional statistical review is needed.

**Were you able to directly test the methods?**

No.

**Comments to author:**

In this manuscript, Janssens et.al., described CUT&Tag2in1, an extension to the very popular CUT&Tag method developed in their lab, to simultaneously profile Pol II-associated active chromatin regions and H3K27me3-associated repressive regulome in single cells. Probing both active and repressive chromatin states in single cells is important for dissecting the underlying regulatory programs of different cell types and is especially demanded in the field of developmental biology and epigenetics. CUT&Tag2in1 provides a simple solution to deconvolute both active and repressive chromatin regions from a single sequencing readout. The manuscript is well written and the methods&analysis details are sufficient enough to allow the community to adopt it to various of single-cell platforms.

My major concern on the current manuscript is about the signal classification to PolS5p and K27me3 reads in single cells:

The authors showing using low salt buffer give sharp peaks compared to CUT&Tag. Did they compare H3K27me3 peak widths under CUT&Tag and CUTAC buffers? In Additional files 1b &c, although H3K27me3 showing much larger fragment lengths compared to PolS5P, but there is still a significant fraction of K27me3 fragments that are <120bp. I believe the impact of this part is minimal to the overall signal, but a systematic illustration of the contribution from this part to the final deconvoluted peaks will be beneficial.

The author utilized fragment length distributions and positional dependencies to deconvolute PolS5p and K27me3 reads from single-cells, which is slightly different from what they used in the bulk analysis. Did the author compare the result from 2for1separator and the result of treating the aggregated single-cell reads as a pseudobulk sample?

The author also found peaks overlapped between H3K27me3/PolS5P. It will be interesting to look into the overlapping peaks with a single cell (both short and long fragments in the same genomic region of a cell) and across cells (short fragment in a subset of cells and long fragments in another), will there be a difference between hESC and K562 as ES cells are keeping bivalent elements.

In addition, there are a few minor points:

1, The authors used heatmaps to show the signals around peaks, these are very informative. However, the number of peaks should also be indicated along with the heatmaps.

2, Fig. S2 ab, the color in the figure is different from those in figure legends.

3, Fig. S3, what is the reproducibility between replicate 1 & 2; some analyses such as co-embedding and overlap of peaks inferred will be useful.

4, Additional file 4 is only mentioned in the method section. What are the numbers of overlapped peaks? This information will be useful to include in the main text.

**Reviewer 2**

**Were you able to assess all statistics in the manuscript, including the appropriateness of statistical tests used?**

No.

**Were you able to directly test the methods?**

No.

**Comments to author:**

This manuscript demonstrated a new technique, CUT&Tag2for1, for profiling both active and repressive chromatin regions in bulk and single cells. Through a deconvolution strategy, the authors leverage fragment length distribution, cut-site densities, and feature width to infer reads mapping to active/repressive chromatin regions. The technique and computation strategy could help analyze chromatin landscape in a large number of samples or cells. However, some essential information and replicates are missing for validation of the method, as discussed below:

1. In the first figure, the authors claim that Pol2S5p-CUTAC maps accessibility of promoters/enhancers similar with H3K4me2 CUTAC and ATAC-seq. To evaluate this, the author should systematically assess the intersection of peaks recovered from the three assays. In addition, the quantitative correlation between different groups should be calculated to support the claim.

2. Additional figure 1b shows that a substantial fraction of Pol2S5p CUTAC signals fall into the large fragment section. This fraction should be quantified. Do these long fragments map to the same genomic locations of the short reads (e.g., any significant bias in promoters/enhancers)?

3. Similarly, a fraction of the H3K27me3 CUTAC signals has a short fragment length distribution based on additional figure 1b. How do these "contaminating" reads affect the signal purity in the bulk analysis?

4. The computation strategy, 2for1separator, utilizes three features to deconvolute the reads from CUT&Tag2for1: fragment length distribution, cut-site densities, and feature width. Is fragment length still the critical feature to separate reads from different chromatin states?

5. Another potential concern for using this technique is that fragment length distribution could vary a lot depending on the enzyme concentration/activity. Biological replicates are needed to evaluate the robustness of the method. Meanwhile, additional figure 2a shows different replicates exhibit different fragment length distributions. Would this affect the deconvolution result for bulk and single cells?

**Reviewer 3**

**Were you able to assess all statistics in the manuscript, including the appropriateness of statistical tests used?**

No.

**Were you able to directly test the methods?**

No.

**Comments to author:**

In the manuscript "Simultaneous CUT&Tag profiling of the accessible and silenced regulome in single cells", Janssens et al. describe a modified CUT&Tag protocol in which two antibodies targeting Pol2 Serine-5 phosphate and H3K27me3 are used, with low salt concentration (as in CUTAC). They also describe a computational pipeline to deconvolute the data, and the development of a single cell version of the method, scCUT&Tag2for1. While this is one of the first reports describing simultaneously application of CUT&Tag for 2 modalities, the manuscript appears to me preliminary and it is not clear to me the advantages of method to CUT&Tag against the individual targets. The manuscript needs major revisions in several points, which I consider precludes publication in Genome Biology in the current form:

- The major principle for the deconvolution, the different sizes associated with Pol2S5P and H3K27me3 is very neat and ingenious. However, would it be applicable to other histone modifications, for instance H3K36me3 combined to H3K27ac? Or would this constitute a limitation of the method? The authors briefly discuss this, but this is an important point to address in the current manuscript.

- From the deconvolution in Figure 3 c and d, it is clear that some of the deconvoluted signal from Pol2S5P originates from H3K27me3 and vice versa, since there is no signal when Pol2S5p/H3K27me3 is run individually. This brings the question on whether the deconvolution of overlapping peaks is efficient. Since one of the major features of the data could be the resolution of these overlaps, to determine if they are biologically relevant, it would be important that the authors dedicate more efforts on this aspect, with further analysis, additional metrics and QC, etc.

Also, small fraction (5-10%) of peaks (Figure 3E,3F) seem to be mis-classified even after omitting the overlapping peaks. Summary statistics of overlapping/mis-classified peaks should also be presented in the main figures.

- In Figure 4, the authors are able to use scCUT&Tag2for1 to distinguish the identity of 2 cell lines. However, it is not clear to me what would be the major advantage of this method, compared to applying scCUT&Tag for Pol2S5P or H3K27me3 separately. Most likely, one would be able to distinguish these cell lines by applying only one of the modalities (these experiments that need to be included in the manuscript as reference). In order to examine the strengths and weakness of scCUT&Tag2for1, application to complex tissues (such as tumour biopsy or mouse tissue) would be required.

- It is not clear to me why the authors first aggregate the reads into pseudobulk and only then apply the 2for1separator algorithm. To me, this defies the purpose of running the experiment in a single cell mode, what would be the caveats of applying the 2for1separator algorithm directly to the single cell data?

- The authors use Gaussian Processes (GP) to model cut-site densities, which takes in account neighbourhood cuts. Will this produce a bias in the deconvolution and could this strategy lead to misassignment of peaks? Could the authors provide evidence that including the neighbouring cuts in the model improves the classifier?

**Authors Response**

**Point-by-point responses to the reviewers’ comments:**

Reviewer #1:

In this manuscript, Janssens et.al., described CUT&Tag2in1, an extension to the very popular CUT&Tag method developed in their lab, to simultaneously profile Pol II-associated active chromatin regions and H3K27me3-associated repressive regulome in single cells. Probing both active and repressive chromatin states in single cells is important for dissecting the underlying regulatory programs of different cell types and is especially demanded in the field of developmental biology and epigenetics. CUT&Tag2in1 provides a simple solution to deconvolute both active and repressive chromatin regions from a single sequencing readout. The manuscript is well written and the methods&analysis details are sufficient enough to allow the community to adopt it to various of single-cell platforms.

*Response: We thank Reviewer #1 for this positive evaluation.*

My major concern on the current manuscript is about the signal classification to PolS5p and K27me3 reads in single cells:

The authors showing using low salt buffer give sharp peaks compared to CUT&Tag. Did they compare H3K27me3 peak widths under CUT&Tag and CUTAC buffers? In Additional files 1b &c, although H3K27me3 showing much larger fragment lengths compared to PolS5P, but there is still a significant fraction of K27me3 fragments that are <120bp. I believe the impact of this part is minimal to the overall signal, but a systematic illustration of the contribution from this part to the final deconvoluted peaks will be beneficial.

*Response: We have added the fragment length distribution for H3K27me3 in high-salt buffer to Additional file 1c. As Reviewer #1 anticipated, the impact is minimal, slightly increasing the fraction of mononucleosomal fragments relative to <120 bp fragments for both K562 and H1 ES cells.*

The author utilized fragment length distributions and positional dependencies to deconvolute PolS5p and K27me3 reads from single-cells, which is slightly different from what they used in the bulk analysis. Did the author compare the result from 2for1separator and the result of treating the aggregated single-cell reads as a pseudobulk sample?

*Response: The deconvolution for single-cell data was performed by pooling the single-cell reads as pseudobulk. 2for1separator computes the target likelihood for each cut and so retains single-cell resolution during deconvolution with the pseudo-bulk only informing the marginal distributions. We have now clarified this in the main text (lines 158-9).*

The author also found peaks overlapped between H3K27me3/PolS5P. It will be interesting to look into the overlapping peaks with a single cell (both short and long fragments in the same genomic region of a cell) and across cells (short fragment in a subset of cells and long fragments in another), will there be a difference between hESC and K562 as ES cells are keeping bivalent elements.

*Response: We thank Reviewer #1 for raising this point. As described in response to Reviewer #2 point 3, overlapping H3K27me3/PolS5P small fragments can be from either or both epitopes in both cell types, which complicates interpretation of bivalency. At the single-cell level CUT&Tag2for1 data is unfortunately too sparse to draw firm conclusions about bivalent domains.*

In addition, there are a few minor points:

1, The authors used heatmaps to show the signals around peaks, these are very informative. However, the number of peaks should also be indicated along with the heatmaps.

*Response: The numbers of sites (TSSs, Enhancers and CUTAC peaks) are now shown in Figure 1.*

2, Fig. S2 ab, the color in the figure is different from those in figure legends.

*Response: The figure and figure legend are concordant.*

3, Fig. S3, what is the reproducibility between replicate 1 & 2; some analyses such as co-embedding and overlap of peaks inferred will be useful.

*Response: We now provide a revised Additional file 3 (New Additional file 4) in which we test the co-embedding of 2 independent replicates as well as the ability to separate cell types in one replicate based on the deconvolution of the other. We find that indeed K562 cells from two independent replicates cluster nicely together according to either the deconvolved Pol2S5p or H3K27me3 signal and away from a second cluster comprised of the H1-hESCs from two independent replicates. In addition we find the deconvolved Pol2S5p or H3K27me3 features defined by one replicate are sufficient to faithfully cluster cells run as an independent replicate.*

4, Additional file 4 is only mentioned in the method section. What are the numbers of overlapped peaks? This information will be useful to include in the main text.

*Response: We observed 139 of 10,661 H1 peaks and 104 of 11,111 K562 peaks in H1-hESC and K562 cell lines respectively. We have now clarified this in the text (lines 464-6).*

Reviewer #2:

This manuscript demonstrated a new technique, CUT&Tag2for1, for profiling both active and repressive chromatin regions in bulk and single cells. Through a deconvolution strategy, the authors leverage fragment length distribution, cut-site densities, and feature width to infer reads mapping to active/repressive chromatin regions. The technique and computation strategy could help analyze chromatin landscape in a large number of samples or cells. However, some essential information and replicates are missing for validation of the method, as discussed below:

*Response: We thank Reviewer #2 for pointing out the missing information, which is now provided.*

1. In the first figure, the authors claim that Pol2S5p-CUTAC maps accessibility of promoters/enhancers similar with H3K4me2 CUTAC and ATAC-seq. To evaluate this, the author should systematically assess the intersection of peaks recovered from the three assays. In addition, the quantitative correlation between different groups should be calculated to support the claim.

*Response: We agree that a systematic evaluation of the concordance between the two CUTAC methods and ATAC-seq is warranted. Accordingly, we have called peaks from ATAC_ENCODE data and used heat map alignments to determine the overlap of CUTAC datasets. Based on these heat maps, we find that when fragments are sampled down to 3.2 million reads, 98-99% of Pol2S5p- and H3K4me2-CUTAC ≤120 bp fragments are centered over ENCODE ATAC-seq peaks, whereas an ENCODE ATAC-seq replicate shows 93% overlap (New Additional file 2b-d). We attribute the better overlap of CUTAC to the ENCODE ATAC-seq standard than a replicate of the same standard to the better resolution of CUTAC over ATAC-seq, with much sharper peaks and better signal-to-noise (see example in the “Response to Reviewers” Supplementary Materials file) for a bidirectional housekeeping gene promoter), so that much more of the CUTAC signal is present in ≤120 bp fragments and is better centered over the midpoint of each accessible site. This close correspondence is confirmed by correlation analysis (New Additional File 2e).*

2. Additional figure 1b shows that a substantial fraction of Pol2S5p CUTAC signals fall into the large fragment section. This fraction should be quantified.

*Response: Fragment size percentages are now shown for both Pol2S5p and H3K27me3 in Figure 1c.*

Do these long fragments map to the same genomic locations of the short reads (e.g., any significant bias in promoters/enhancers)?

*Response: Yes. The large (>120 bp) fragments are centered over the small (≤120 bp) fragments, as can be seen in the aligned tracks at a representative region (new Additional File 2a) and in the genome-wide heatmaps of signal over ENCODE ATAC-seq sites (New Additional File 2b-d).*

3. Similarly, a fraction of the H3K27me3 CUTAC signals has a short fragment length distribution based on additional figure 1b. How do these "contaminating" reads affect the signal purity in the bulk analysis?

*Response: Reviewers #1 and #3 made similar points, and in response we now show the length distribution for H3K27me3 under 300 mM CUT&Tag tagmentation conditions in Additional file 1b-c. For K562 cells, we did not observe a difference (13% of fragments for 10 mM tagmentation and 14% for 300 mM tagmentation) although 25% “contamination” was observed when 10% 1,6-hexanediol was present during tagmentation. We had previously shown that H3K27me3 CUT&Tag with tagmentation in low salt results in a low level of integration precisely at accessible sites [Kaya-Okur et al. (2020) Nature Protocols 13:3264-83 Figure 5a]. However, the large majority of accessible site labeling in a CUT&Tag2for1 experiment results from anchoring to the Pol2S5p epitope. Since the sites of Tn5 integration are precisely the same, the “contaminating” integrations provide a small positive contribution to the resulting profile. In this way, the 2for1 strategy turns what would otherwise be an artifact into a desirable feature, and we now make this point in the text (lines 194-5).*

4. The computation strategy, 2for1separator, utilizes three features to deconvolute the reads from CUT&Tag2for1: fragment length distribution, cut-site densities, and feature width. Is fragment length still the critical feature to separate reads from different chromatin states?

*Response: We have now added new Additional file 3 to demonstrate the importance of fragment length distributions and cut-site densities to the deconvolution (See Appendix below for further details). We show that the sensitivity and accuracy of deconvolution is reduced if fragment length is not used as one of the features for deconvolution. However, 2for1separator works extremely well even if the prior knowledge about the fragment length distributions of the two antibodies are not known. Thus, the inherent difference in fragment length distributions of the two antibodies used in CUT&Tag2for1 is essential for successful deconvolution but a well-defined prior knowledge about the fragment length distributions is not necessary.*

5. Another potential concern for using this technique is that fragment length distribution could vary a lot depending on the enzyme concentration/activity. Biological replicates are needed to evaluate the robustness of the method. Meanwhile, additional figure 2a shows different replicates exhibit different fragment length distributions. Would this affect the deconvolution result for bulk and single cells?

*Response: We have previously shown that CUT&Tag is inherently insensitive to differences in enzyme concentration for both rare and abundant epitopes (PMID:31036827, Supplementary Figure 5) and to cell number over >2-order-of magnitude range (PMID: 33191916, Figure 1) owing to the use of saturating concentrations and stringent washes after each incubation to remove excess reagent. The tethering of pA-Tn5 to paused PolII is an inherent advantage of CUTAC over ATAC-seq, in which Tn5 enzyme concentration must be scaled to cell number for best results. For the same reasons, CUT&Tag is also robust to differences in antibody concentrations, although we have found that CUT&Tag2for1 is sensitive to the ratio of the two antibodies. To address this potential concern, we compared 2for1separator deconvolution of samples from an experiment that varied the antibody ratios over an 8-fold range and have included the results in the “Response to Reviewers” Supplementary Materials file.*

Reviewer #3:

In the manuscript "Simultaneous CUT&Tag profiling of the accessible and silenced regulome in single cells", Janssens et al. describe a modified CUT&Tag protocol in which two antibodies targeting Pol2 Serine-5 phosphate and H3K27me3 are used, with low salt concentration (as in CUTAC). They also describe a computational pipeline to deconvolute the data, and the development of a single cell version of the method, scCUT&Tag2for1. While this is one of the first reports describing simultaneously application of CUT&Tag for 2 modalities, the manuscript appears to me preliminary, and it is not clear to me the advantages of method to CUT&Tag against the individual targets. The manuscript needs major revisions in several points, which I consider precludes publication in Genome Biology in the current form:

*Response: We thank Reviewer #3 for raising these important issues, which we have resolved as described below.*

- The major principle for the deconvolution, the different sizes associated with Pol2S5P and H3K27me3 is very neat and ingenious. However, would it be applicable to other histone modifications, for instance H3K36me3 combined to H3K27ac? Or would this constitute a limitation of the method? The authors briefly discuss this, but this is an important point to address in the current manuscript.

*Response: We now refer to this as a limitation of the method in the text (lines 201-4). Histone marks such as H3K27ac and H3K36me3 cannot be paired with other histone marks in a CUT&Tag2for1 assay because they both give nucleosome-size fragments and so cannot be efficiently deconvolved. These marks can be paired with Pol2S5p, however, H3K27ac is redundant with Pol2S5p, and we have found that H3K36me3 antibodies give clean results in the presence of 300 mM NaCl, but noisy data under low-salt CUTAC conditions. Although in principle our strategy can be applied to other pairs of epitopes, most researchers doing single-cell epigenomic profiling care about active and silenced enhancers and promoters, which drive development and cancer, and our method maps all of these regulatory elements in the same single cells. Unlike the current generation of multi-omics strategies, ours requires no extra effort over single-cell CUT&Tag, which is rapidly increasing in popularity, and we do not expect much demand for epitopes beyond those that capture the functional active and silent regulomes.*

- From the deconvolution in Figure 3 c and d, it is clear that some of the deconvoluted signal from Pol2S5P originates from H3K27me3 and vice versa, since there is no signal when Pol2S5p/H3K27me3 is run individually. This brings the question on whether the deconvolution of overlapping peaks is efficient. Since one of the major features of the data could be the resolution of these overlaps, to determine if they are biologically relevant, it would be important that the authors dedicate more efforts on this aspect, with further analysis, additional metrics and QC, etc.

*Response: The other reviewers made similar points about the overlap. As we explained in response to Reviewer #2 point 3, The low-salt CUTAC protocol that results in tagmentation of accessible chromatin by Tn5 tethered to H3K4me2/3 or Pol2S5p also causes accessible chromatin sites adjacent to or within H3K27me3 domains to be tagmented at a very low level, and this is why we used 300 mM NaCl to suppress accessible site tagmentation in CUT&Tag (PMIDs: 31036827 and 32913232). We have now clarified this point in the main text (lines 196-7). Therefore, we used standard CUT&Tag conditions with 300 mM NaCl to suppress accessible chromatin tagmentation for our H3K27me3 “ground-truth” control sample. Our peak-calling procedure can accurately handle these signals and minimize the number of misclassified peaks. The number of unresolved overlaps is small, and the general deconvolution is not impaired by these regions. As noted in response to Reviewer 1, we observed only 139 of 10,661 H1 peaks and 104 of 11,111 K562 peaks in H1-hESC and K562 cell lines respectively (lines 485-6).*

Also, small fraction (5-10%) of peaks (Figure 3E,3F) seem to be mis-classified even after omitting the overlapping peaks. Summary statistics of overlapping/mis-classified peaks should also be presented in the main figures.

*Response: We thank the reviewer for highlighting this - the requested statistics are now included for the main figures. We called peaks from the single antibody data for Pol2S5p and H3K27me3. Assuming that this reflects the ground truth, we call a peak misclassified only if it overlaps with a single antibody peak of the other target. We used fragments of length > 147 bp for calling H3K27me3 peaks, since the true targets produce reads that are protected by nucleosomes (~150 bp). Pol2S5P peaks were called using MACS2 and H3K27me3 peaks were called using SEACR.*

- In Figure 4, the authors are able to use scCUT&Tag2for1 to distinguish the identity of 2 cell lines. However, it is not clear to me what would be the major advantage of this method, compared to applying scCUT&Tag for Pol2S5P or H3K27me3 separately.

*Response: A major advantage of scCUT&Tag2for1 is that we obtain complementary regulomes from each single cell, not possible using single modalities. Single-cell multi-modal methods, such as ATAC-seq + RNA-seq are gaining in popularity, but these require complex workflows and the information gained about regulation is somewhat redundant, as both reflect gene expression. In contrast, scCUT&Tag2for1 provides both gene expression (chromatin accessibility defined by paused RNA polymerase II) and Polycomb developmental silencing, independent programs that together map the transcriptional regulome in each single cell. The single-cell analyses that we provided in Figure 4 demonstrate that enhancer/promoter marks (PolIISer5p) and Polycomb domain marks (H3K27me3) from the same single cells are each characteristic of different cell types by UMAP and heatmap analyses, so it is indeed the case that either one suffices to separate these two cell types.*

Most likely, one would be able to distinguish these cell lines by applying only one of the modalities (these experiments that need to be included in the manuscript as reference). In order to examine the strengths and weakness of scCUT&Tag2for1, application to complex tissues (such as tumour biopsy or mouse tissue) would be required.

*Response: We do not claim that de novo separation of cell types by clustering is a major advantage of our method, because in principle the additional information is less than or equal to the information obtained from the more informative of the two epitopes. Rather, the major motivation for developing our method is to distinguish active and silenced genomic regions within a cell. For example, in our recent application of automated and single-cell CUT&Tag to characterizing mixed-lineage leukemia patient samples, we observed surprising heterogeneity for H3K27me3 at sites of cancer driver genes between cells within a UMAP cluster (Janssens et al. Nature Genetics 2021 Nov;53(11):1586-1596, see Figure 3i-j, e.g. HoxA9 versus Meis1). In this earlier paper we had exploited matching clusters from different single-cell profiling experiments to infer the variation in the two marks within a cell type. However, the scCUT&Tag2for1 strategy we develop here directly determines active and repressed regions in the same individual cells.*

- It is not clear to me why the authors first aggregate the reads into pseudobulk and only then apply the 2for1separator algorithm. To me, this defies the purpose of running the experiment in a single cell mode, what would be the caveats of applying the 2for1separator algorithm directly to the single cell data?

*Response: Unlike RNA-seq, where the amplification of transcription results in high fragment counts limited to the most highly expressed genes, for single-cell chromatin profiling there are only two copies of each genomic locus per diploid cell. Furthermore, Pol2S5p CUTAC peaks are mostly ~1 fragment wide, so that even with 100% efficiency true positive signals would be equivalent to background. Therefore what Reviewer 3 is asking for is not feasible, nor is it necessary to achieve single-cell resolution with 2for1separator. Rather, methods designed for single-cell chromatin data, such for ArchR (PMID: 33633365), first create a pseudo-bulk profile by aggregating the single-cell data, and we follow a similar approach. Then 2for1separator computes the target likelihood for each cut and so retains single-cell resolution during deconvolution with the pseudo-bulk only informing the marginal distributions. Following deconvolution, fragment count per peak is computed for each cell and used for downstream analysis such as clustering and visualization, again consistent with what is routinely done in the single-cell field.*

- The authors use Gaussian Processes (GP) to model cut-site densities, which takes in account neighbourhood cuts. Will this produce a bias in the deconvolution and could this strategy lead to misassignment of peaks? Could the authors provide evidence that including the neighbouring cuts in the model improves the classifier?

*Response: Neighborhood information is only shared within a target-specific marginal and should not adversely impact the deconvolution accuracy. In response to Reviewer #2 point 4, we have added New Additional file 3 to demonstrate the importance of fragment length distribution and cut-site densities to the deconvolution: Not encoding information about neighboring cuts through the GP covariance severely degrades the deconvolution, highlighting the importance of neighboring cuts for classification performance (see the Appendix in the “Response to Reviewers” Supplementary Materials file).*

**Second round of review**

**Reviewer 1**

The authors have addressed my concerns in the revised manuscript. The community can benefit from the clear demonstrations of both features and limitations of this methods in the revised version.

**Reviewer 2**

The authors have satisfactorily resolved all my comments. I suggest the acceptance of the manuscript.

**Reviewer 3**

While the method is very interesting, I am afraid that the authors only address my comments in a superficial manner, with important metrics still missing. Moreover, the limited application to other combinations of histone modifications is an aspect that should be explored and discussed in more detail in the manuscript. The revision does not alter my assessment that the manuscript is still preliminary. The authors highlight that the major advance in the paper is the possibility of performing repressive and active histone mark analysis in the same cell and not to separate cell types by clustering, but this aspect is not explored in sufficient extent in the current manuscript, to determine for instance if the method allows to unveil repressive substates, as the authors suggest in their response.

**Authors Response**

**Point-by-point responses to the reviewers’ comments:**

Reviewer #1:

The authors have addressed my concerns in the revised manuscript. The community can benefit from the clear demonstrations of both features and limitations of this methods in the revised version.

Reviewer #2: The authors have satisfactorily resolved all my comments. I suggest the acceptance of the manuscript.

*Response: We thank Reviewers #1 and #2 for these positive assessments of our revised manuscript.*

Reviewer #3:

“While the method is very interesting, I am afraid that the authors only address my comments in a superficial manner, with important metrics still missing. Moreover, the limited application to other combinations of histone modifications is an aspect that should be explored and discussed in more detail in the manuscript. “

*Response: On this point, Reviewer #3 had asked us to answer the following questions: “However, would it be applicable to other histone modifications, for instance H3K36me3 combined to H3K27ac? Or would this constitute a limitation of the method?” In our response, we had explained that our method cannot be used for multiple histone modifications because they give nucleosome-sized fragments, and so no size discrimination is possible. Accordingly, we had expanded the text to clarify this point: “A limitation of our deconvolution strategy is that it requires a large fragment/large feature and small fragment/small feature pair for best performance.” In other words, what Reviewer #3 was asking for is not possible, and so we answered the questions by conceding in the text that this is a limitation of the method. We have now expanded this statement to make it clearer that, whereas profiling multiple histone modifications is not possible, our strategy can apply to other combinations of chromatin features. Also, to better emphasize these points, we have moved them to the Conclusions section as a limitation of the method.*

*From a user’s perspective, this is at best a very minor issue. Like ATAC-seq, CUTAC provides accessibility data, and we show in Figure 1c and Additional file 1a that PolIISer5 CUTAC outperforms ATAC-seq from the ENCODE project for promoter/enhancer mapping in sensitivity (# of peaks called), specificity (Fraction of reads in peaks) and resolution. ATAC-seq, with 4620 citations in 2021 alone (according to Google Scholar) is rapidly replacing ChIP-seq for chromatin profiling, despite the fact that ChIP-seq can profile any chromatin component whereas ATAC-seq profiles only one thing, accessibility. Single-cell ATAC-seq also dominates for single-cell chromatin profiling, but can only map active regulatory elements, whereas CUT&Tag2for1 adds mapping of silenced regulatory elements in the same experiment with no additional steps. In this context, we do not think that the ability to extend CUT&Tag2for1 to other chromatin marks is an issue that prospective users of the method will be concerned about.*

“The revision does not alter my assessment that the manuscript is still preliminary. The authors highlight that the major advance in the paper is the possibility of performing repressive and active histone mark analysis in the same cell and not to separate cell types by clustering, but this aspect is not explored in sufficient extent in the current manuscript, to determine for instance if the method allows to unveil repressive substates, as the authors suggest in their response.”

*Response: On this point, Reviewer #3 had asked us to address the following issue: “Most likely, one would be able to distinguish these cell lines by applying only one of the modalities (these experiments* that *need to be included in the manuscript as reference).” We had addressed this issue by pointing out that the ability to distinguish both active and silenced regulatory elements in the same cell provides value in itself, for example in detecting lineage plasticity in cancer cells.*

*Since the major advance of CUT&Tag2for1 was made possible by our invention of chromatin accessibility mapping based on paused RNA Polymerase II (Pol2S5p CUTAC), which provides higher data quality than ENCODE ATAC-seq data, we now emphasize the importance of this advance in the Discussion. Indeed, we consider our demonstration that Pol2S5p CUTAC maps chromatin accessibility better than ATAC-seq to be the best and most direct evidence that chromatin accessibility measured by many different assays over the past nearly half-century is driven by transcription.*
